# Transcriptome and Proteome Data Reveal Candidate Genes for Pollinator Attraction in Sexually Deceptive Orchids

**DOI:** 10.1371/journal.pone.0064621

**Published:** 2013-05-29

**Authors:** Khalid E. M. Sedeek, Weihong Qi, Monica A. Schauer, Alok K. Gupta, Lucy Poveda, Shuqing Xu, Zhong-Jian Liu, Ueli Grossniklaus, Florian P. Schiestl, Philipp M. Schlüter

**Affiliations:** 1 Institute of Systematic Botany & Zürich-Basel Plant Science Centre, University of Zurich, Zürich, Switzerland; 2 Functional Genomics Centre Zurich, Zürich, Switzerland; 3 Institute of Plant Biology & Zürich-Basel Plant Science Centre, University of Zurich, Zürich, Switzerland; 4 Centre for Model System Proteomes, Zürich, Switzerland; 5 Shenzhen Key Laboratory for Orchid Conservation and Utilization, The Orchid Conservation & Research Centre of Shenzhen, Shenzhen, China; 6 The Centre for Biotechnology and BioMedicine, Tsinghua University, Shenzhen, China; 7 College of Forestry, South China Agricultural University, Guangzhou, China; Chiba University, Japan

## Abstract

**Background:**

Sexually deceptive orchids of the genus *Ophrys* mimic the mating signals of their pollinator females to attract males as pollinators. This mode of pollination is highly specific and leads to strong reproductive isolation between species. This study aims to identify candidate genes responsible for pollinator attraction and reproductive isolation between three closely related species, *O. exaltata*, *O. sphegodes* and *O. garganica*. Floral traits such as odour, colour and morphology are necessary for successful pollinator attraction. In particular, different odour hydrocarbon profiles have been linked to differences in specific pollinator attraction among these species. Therefore, the identification of genes involved in these traits is important for understanding the molecular basis of pollinator attraction by sexually deceptive orchids.

**Results:**

We have created floral reference transcriptomes and proteomes for these three *Ophrys* species using a combination of next-generation sequencing (454 and Solexa), Sanger sequencing, and shotgun proteomics (tandem mass spectrometry). In total, 121 917 unique transcripts and 3531 proteins were identified. This represents the first orchid proteome and transcriptome from the orchid subfamily Orchidoideae. Proteome data revealed proteins corresponding to 2644 transcripts and 887 proteins not observed in the transcriptome. Candidate genes for hydrocarbon and anthocyanin biosynthesis were represented by 156 and 61 unique transcripts in 20 and 7 genes classes, respectively. Moreover, transcription factors putatively involved in the regulation of flower odour, colour and morphology were annotated, including Myb, MADS and TCP factors.

**Conclusion:**

Our comprehensive data set generated by combining transcriptome and proteome technologies allowed identification of candidate genes for pollinator attraction and reproductive isolation among sexually deceptive orchids. This includes genes for hydrocarbon and anthocyanin biosynthesis and regulation, and the development of floral morphology. These data will serve as an invaluable resource for research in orchid floral biology, enabling studies into the molecular mechanisms of pollinator attraction and speciation.

## Introduction

The orchids (Orchidaceae) are one of the most species-rich plant families, and their remarkable floral diversity and pollination biology have long fascinated evolutionary biologists [1], [2]. It has been estimated that about one third of orchids are pollinated by deception, i.e. without rewarding their pollinators [2], [3]. For example, *Ophrys* L., a Euro-Mediterranean genus of sexually deceptive orchids, is mostly pollinated by male insects, primarily solitary bees [4]. These orchids mimic the visual, tactile, and olfactory signals of the females of their pollinators, so that male insects are attracted and try to copulate with the flower labellum (a modified petal). During these so-called ‘pseudo-copulations’ the pollinia (pollen packets) become attached to the bees and are transferred during subsequent visits of the males to other flowers [5], [6], [7]. Numerous behavioural studies have shown that the *Ophrys*-pollinator relationship is highly specific: each *Ophrys* species is usually pollinated by only one (or very few) insect species [4], [5], [6], [8], [9], [10]. It has also been shown that floral odour is the key factor in attracting specific pollinators and eliciting male mating behaviour [11], [12], [13]. In addition to odour, flower colour (including UV) and morphology (shape, size and texture) including epidermal structure (e.g. trichomes) also contribute to successful pollination [4], [14], [15], [16], [17]. Nonetheless, colour signals are of less importance than floral odour in a group of solitary bee-pollinated species [18] similar to those analysed in the present study.

In *Ophrys* orchids, floral odour mimics the sex pheromone produced by the female of the pollinators [11], [12], [19]. This pseudo-pheromone is a mixture of cuticular alkane and alkene hydrocarbons produced by the flower labellum: specifically alkanes (saturated straight-chain hydrocarbons) with different carbon chain length (C_21_-C_31_) and alkenes (monounsaturated hydrocarbons) that can additionally vary in their *cis*-double-bond positions (e.g. 7-, 9-, or 12-) [17], [20]. The relative amounts of alkanes and alkenes differ significantly among *Ophrys* species, thereby producing different pseudo-pheromone odour bouquets that attract different species of male bees as their pollinators [13], [21], [22]. These hydrocarbons are therefore crucial for pollinator-mediated reproductive isolation among *Ophrys* species [22], and thus play an important role in pollinator-mediated speciation in these orchids [20], [21], [23], [24].

Because of their strong pollinator-mediated reproductive isolation and the relatively well-understood chemical ecology of their highly specific pollination, *Ophrys* orchids provide an excellent system for studying pollinator-driven speciation and for identifying reproductive ‘barrier genes’ [17], [25], that is, genes directly involved in reproductive isolation [26]. Three closely related and sympatric *Ophrys* species, *O. exaltata, O. sphegodes* and *O. garganica* (Fig. S1) are investigated in this study. They are genetically compatible and crossable, but are strongly isolated from each other by pollinator-mediated, odour-based reproductive isolation, whereas post-pollination reproductive barriers are weak [22]. These species produce different odour bouquets: *O. exaltata* produces high levels of 7-alkenes, whereas *O. sphegodes and O. garganica* produce high levels of 9- and 12-alkenes in different proportions and carbon chain lengths [22]. Therefore, genes underlying these floral odour differences are candidate barrier genes, or possibly even speciation genes, among the study species. Alkanes and alkenes are expected to be derived from very-long-chain fatty acid (VLCFA) biosynthesis in epidermal cells of the flower labellum [17], [20], [27], [28]. Although acyl-ACP (acyl carrier protein) desaturases that introduce a double-bond into alkene precursors have previously been identified as barrier genes among *O. sphegodes* and *O. exaltata* [20], [24], other genes responsible for odour differences, such as hydrocarbon chain length differences among *O. sphegodes* and *O. garganica*, are still unknown.

The study of candidate genes in *Ophrys* orchids has so far been hindered by the lack of sequence resources. Currently, there is no genome sequence publically available for any orchid, and there are no comprehensive genome, transcriptome, or proteome resources for sexually deceptive orchids. A small number of orchid expressed sequence tags (ESTs) obtained by Sanger sequencing are available [29], [30], [31], [32], including 277 ESTs from *Ophrys* [33]. Although transcriptomes of the *Phalaenopsis* and *Oncidium* ‘Gower Ramsey’ orchids have recently been released [34], [35], these orchids are from the subfamily Epidendroideae and are only distantly related to *Ophrys* (subfamily Orchidoideae). Recently, next-generation sequencing such as 454 pyrosequencing has been widely used for *de novo* sequencing and EST analyses. These technologies have proven effective for expanding the available sequence information not only for model species [36], [37] but also for non-model species [38], [39] such as *Ophrys*, the large genome size of which (∼10 Gbp [40]) makes transcriptome sequencing a good choice for gene discovery. Moreover, shotgun proteomics by tandem mass spectroscopy (MS/MS) has recently been successfully used for the discovery of the protein components of various biological systems for which no prior information was available [41].

The current study aims to aid progress in orchid biology by (1) uncovering candidate genes for specific pollinator attraction and pollinator-mediated reproductive isolation among three *Ophrys* species, (2) providing a benchmark reference transcriptome from the orchid subfamily Orchidoideae, and (3) providing the first proteomic data for orchids. We address these questions by use of a systems biology approach, combining high throughput next-generation sequencing technologies (454 pyrosequencing, together with Solexa and Sanger sequencing) and MS/MS shotgun proteomics, in three sexually deceptive *Ophrys* species.

## Results and Discussion

### Sequencing and Assembly

**Figure 1:**
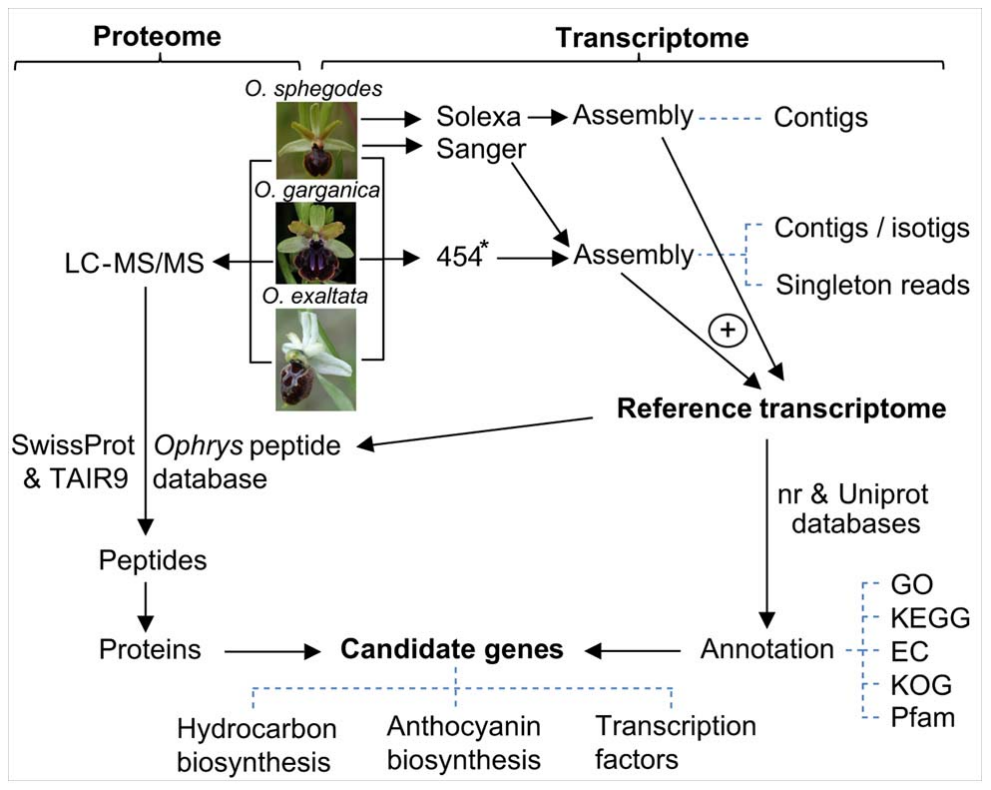
Flow chart of transcriptome and proteome analysis. Labellum tissue from mature, unpollinated flowers was used, except where marked by an asterisk (*), indicating that additional material from sepals, petals, leaves, bracts, and flower buds was included.

**Figure 2:**
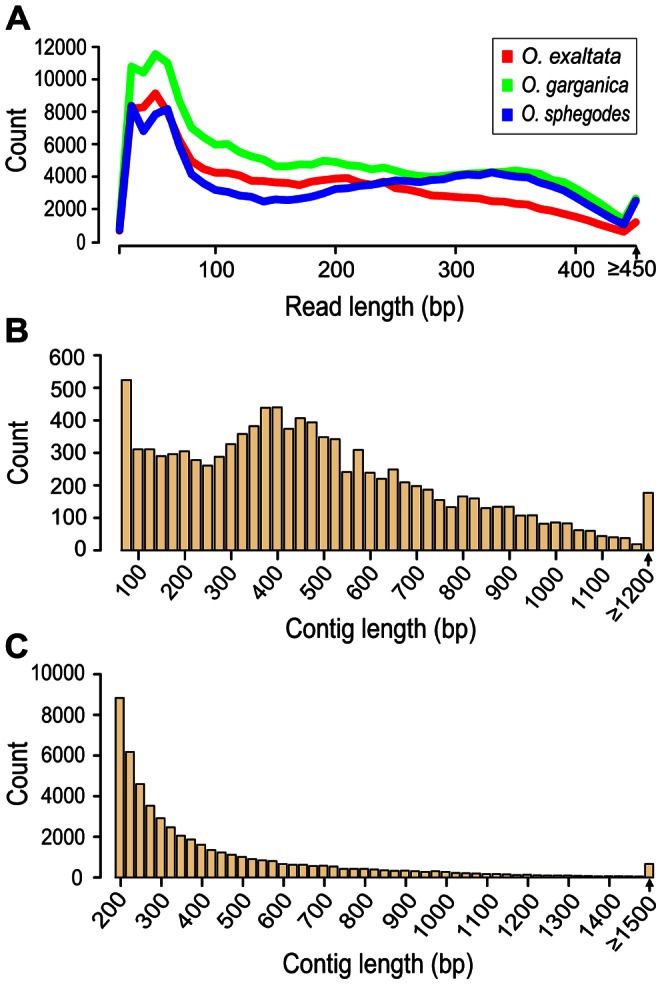
Sequence length distributions. *(A)* Distribution of 454 read lengths after filtering and adapter removal for the three *Ophrys* species. *(B)* Contig length distribution (20 bp windows) for the pooled 454 assembly of the three *Ophrys* species. *(C)* Distribution of contig/isotig lengths in the *Ophrys* reference transcriptome (20 bp windows).

Three normalised cDNA libraries were constructed from three different *Ophrys* species, *O. exaltata*, *O. garganica*, and *O. sphegodes*. For all three libraries, different tissues (mostly of floral origin) were pooled (see Materials and Methods; Fig. 1). These libraries were 454 pyrosequenced, resulting in a total of 71.3 Mbp of sequence data after processing (Table S1), with approximately 80% of the reads between 100 and 450 bp in length (Fig. 2A). All the high quality reads generated in this study are available in the Sequence Read Archive (SRA) of the National Centre for Biotechnology Information (NCBI) with the accession number SRA060767. Additional sequencing of *O. sphegodes* flower labella yielded 1.7 Mbp of Sanger (dbEST library LIBEST_028084; dbEST IDs 77978749-77979571; GenBank accessions JZ163765-JZ164587) and 2.5 Gbp of Illumina Solexa (SRA060767) data (Table S1).

**Figure 3:**
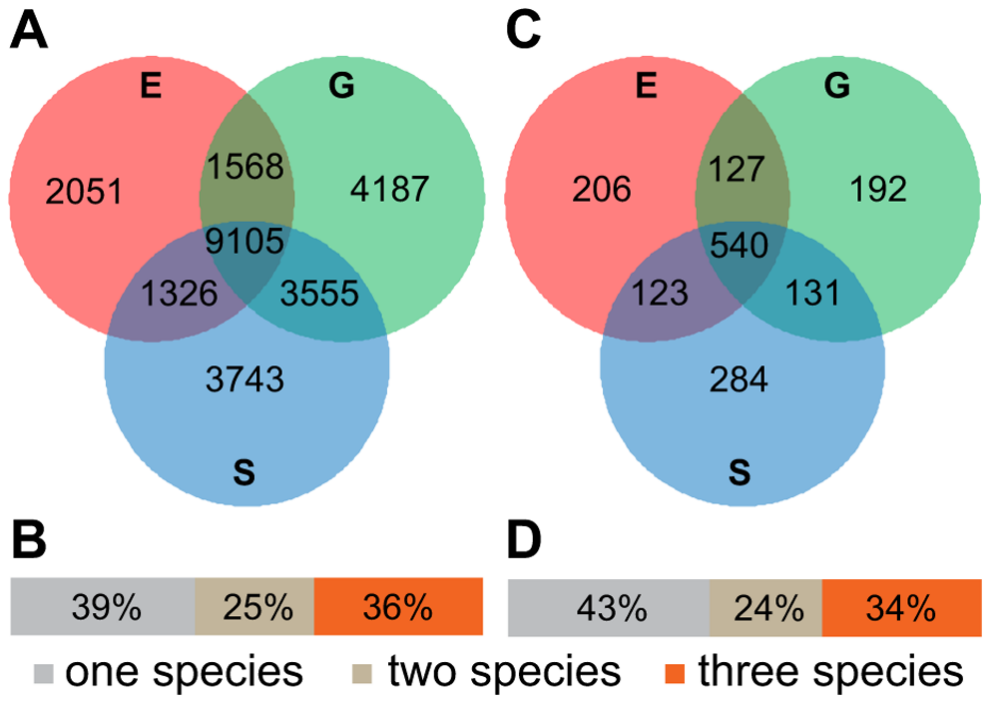
Overlap of transcriptome and proteome data among three orchid species. *(A)* Venn diagram showing the species overlap in 454 reads mapped back onto the reference transcriptome. *(B)* Bar graph indicating the extent of read sharing. *(C)* Venn diagram showing the overlap of *Ophrys* proteomes (HQ data of proteins with corresponding transcripts). *(D)* Bar graph indicating the extent of proteome overlap among species. *(A,C)* E, *O. exaltata*; G, *O. garganica*; S, *O. sphegodes*.

Solexa reads were assembled into contigs, whereas 454 and Sanger reads of the three species were separately assembled into isotigs (transcripts) (Table 1; Fig. 2B), thereby retaining putative gene/transcript relationships. This process left unassembled high-quality singleton reads, which can be considered to be either rare transcripts or artefacts. However, cross-validation of singleton reads (Table 2) by mapping of Solexa data suggested that the majority of singleton reads are not artefacts but represent real transcripts. Pooled 454 and Sanger assembly of all species together increased the effectiveness of the assembly (Table 1). Also, hybrid assemblies combining short (e.g. Solexa) and long (454 or Sanger) reads can improve *de novo* assembly of genomes and transcriptomes (for average read lengths, see Table S1) [42], [43], and short reads can improve coverage and the mining for lowly expressed genes [42]. Therefore, our pooled 454 and Sanger assembly was further merged with the *O. sphegodes* Solexa assembly to produce a final assembly, which we refer to as the *Ophrys* reference transcriptome (Table 1). Overall, this assembly contained 51 795 contigs and isotigs, with an average contig/isotig length of 441 bp, and 70 122 singleton reads with an average length of 285 bp (Table 1, Fig. 2C). The proportion of transcripts shared among species was determined by mapping the 454 reads of each species to the assembled contigs and isotigs present in the reference transcriptome (Fig. 3A). The majority of transcripts were shared among three or at least two species (Fig. 3B), the highest number of transcripts being shared among *O. garganica* and *O. sphegodes* (Fig. 3A), suggesting a closer overall transcriptome similarity among these species, which is consistent with overall floral similarities (see Fig. S1).

**Table 1:** Sequence assembly summary.

	*O. exa.* 454	*O. gar.* 454	*O. sph.* 454+ Sanger	*O. sph.* Solexa	*O. sph.* 454+ Sanger+Solexa	Pooled 454+ Sanger	Reference transcriptome (Pooled 454+ Sanger+Solexa)
Number of contigs/isotigs	2205	4172	3815	50 230	51 465	9375	51 795
Bases in assembly (nt)	1 022 978	2 334 132	1 925 958	19 166 533	20 501 805	5 777 134	22 837 772
Average contig/isotig length (nt)	463	559	504	382	398	616	441
N_50_ length (nt)	498	610	524	394	303	692	322
Longest contig/isotig length (nt)	1860	2777	1811	4273	4273	4771	4780
Number of singleton reads^1^	35 567	42 261	43 228^2^	N/A	43 228	70 122	70 122
Average singleton read length (nt)	273	289	290	N/A	290	285	285

**.**Individual assembly statistics are shown for data from different sequencing technologies (454, Sanger, Solexa) and different species (*O. exaltata*, *O. garganica*, and *O. sphegodes*), where ‘Pooled 454’ refers to 454 data pooled from all three species. N_50_ length denotes the length-weighted median contig length of a given assembly.

1 Singleton reads from 454 and Sanger data only.

2 Out of these, 92 are Sanger reads.

**Table 2:** Cross-validation of NGS data sets.

Reference data	N_ref_	Mapped by	N_map_	%age (mapping)
Solexa-only contigs (*O. sph.*)	42 493	454 reads (3 *spp*.)	16 255	38.3%
454 reads (3 *spp*.) and Sanger singleton reads (*O. sph.*)^1^	25 287 (92)	Solexa reads (*O. sph.*)	15 316 (52)	60.6% (56.5%)
454 singleton reads (*O. exa.*)	18 664	Solexa reads (*O. sph.*)	9752	52.3%
454 singleton reads (*O. gar.*)	26 171	Solexa reads (*O. sph.*)	16 225	62.0%

Summary of the proportion of a given sequence data set to which reads from another NGS data set can be mapped. N_ref_: number of sequences in the reference data set; N_map_: number of sequences in the reference data set that is mapped by the query data set; %age (mapping): N_map_ expressed as a percentage. The term “3 *spp.*” refers to data from all three orchid species.

1 In this row, values in brackets are for Sanger reads.

The combination of the 454 transcriptomes of the three species with the EST Sanger sequencing and Solexa data into a reference transcriptome represents the maximum amount of genetic information available to date for *Ophrys*. In total, 121 917 unique putative *Ophrys* transcripts were obtained and used for annotation and subsequent analysis. This is considerably more than the published *Phalaenopsis* orchid transcriptome (42 863 transcripts) [35], and comparable to that of *Oncidium* [34] in terms of contigs (50 908), although the latter transcriptome had a higher number of singleton reads (120 219). We note that our *Ophrys* reference transcriptome (assembled sequences and annotation provided in Supplementary Files S1 and S2, respectively) was generated mostly from floral tissues. This is the first comprehensive sequence resource both from orchids of the Orchidoideae subfamily, and for sexually deceptive orchids.

### Proteomics Results

To obtain a more comprehensive understanding of *Ophrys* flowers and to further corroborate the authenticity of *Ophrys* transcripts, we performed large-scale shotgun proteomic analysis using MS/MS. Proteins were extracted from labellum tissue of each study species, digested and subjected to liquid chromatography-tandem mass spectrometry (LC-MS/MS) to obtain three proteomics data sets. Mass spectra were searched against SwissProt and *Arabidopsis* TAIR9 protein databases to identify peptides. Additionally, spectra were searched against protein databases created from the *Ophrys* reference transcriptome obtained in this study. Stringent criteria were used for the assignment of spectra to peptides (95% peptide identification probability) in Scaffold 3.3 (Proteome Software Inc., USA). In order to maximise the utility of proteomics data for uncovering proteins predicted by the orchid transcriptome, a minimum of one unique peptide was used for protein identification, while using two different stringency levels for the probabilistic assignment of peptides to proteins (99% for highest quality, HQ; 90% to maximise protein discovery, PD, in the absence of a fully sequenced genome). The proteomics data generated in this study are available from the PRIDE database [44] under accession numbers 27721–27914 and the ProteomeXchange Consortium (http://proteomecentral.proteomexchange.org) under accession number PXD000069 (doi: 10.6019/PXD000069).

A total of 5496 contaminant-free, HQ unique peptides (PD: 7487) were found in the *Ophrys* proteome (Tables 3, S2 and S3). Out of these data, 93.8% of HQ spectra matched 1603 HQ (2644 PD) proteins predicted from *Ophrys* transcriptome data. This demonstrates the high quality of the transcriptome assembly and the necessity of its use for *Ophrys* protein identification. Out of these HQ proteins, 1328 were only found in *Ophrys* transcriptome data (PD: 1880), but not in SwissProt or TAIR9 databases, whereas 16.6% of HQ spectra (275 HQ, 764 PD proteins) also matched proteins in those databases. An additional 204 putative HQ proteins (PD: 887) from 1222 HQ spectra not present in *Ophrys* transcriptome data (Table 3) could be identified using TAIR and SwissProt databases. Using the FunCat classification scheme [45] for spectra with a hit in the TAIR database, no significant difference (*p*>0.01; Fisher’s exact test) in any functional category could be found among the entire protein set and proteins present in the *Ophrys* transcriptome data. No orchid peptides matched translations from different strands of the same transcript (Table 3), further indicating the overall high quality of the assembly.

**Table 3:** Summary of proteomics data.

	Protein discovery (PD)				Highest quality (HQ)			
Statistic	*O. exa.*	*O. gar.*	*O. sph.*	Total	*O. exa.*	*O. gar.*	*O. sph.*	Total
N raw spectra	9676	8419	7840	25 935	7433	6648	6344	20 425
N clean spectra	9357	8181	7588	25 126	7127	6426	6102	19 655
N unique peptides	4072	3835	4137	7487	2932	2878	3164	5496
N proteins (transcriptome)^1^	1748	1672	1610	2644	1201	1222	1206	1603
N proteins (non-transcriptome)^2^	403	405	439	887	130	134	143	204
Total N proteins	2110	1943	2031	3531	1331	1356	1349	1807

Data are presented for species individually and for the total of all three species combined. The protein discovery (PD) analysis was performed at a protein identification threshold of 90%, the highest quality (HQ) data set was compiled at a threshold of 99%. For spectra, numbers are given before (‘raw’) and after (‘clean’) removal of known contaminants. All other numbers were obtained from cleaned spectra only.

1 Proteins matching a sequence in the *Ophrys* reference transcriptome. In the HQ data set, 115 were 454 singleton reads, 4 Sanger singleton reads, 388 Solexa contigs, 343 pyrosequencing isotigs and 753 combined 454/Solexa contigs. Among these 1603 HQ orchid proteins, no peptide matched translations from different strands, and only 11 proteins (0.7%) had peptides matching to two reading frames of the same transcript strand (in each case due to a single frame shift).

2 Proteins not matching any *Ophrys* transcript, but with SwissProt and/or TAIR9 database hit.

Like in other published studies [42], [46], [47], [48], far fewer unique proteins (whether HQ or PD) could be identified than transcripts. Possible reasons include the complexity of the proteome and lower protein coverage as compared to transcript sequence data. The overlap among orchid-specific proteins for the three study species (Fig. 3C,D) was similar to the proportions of shared transcripts among species. All in all, proteomic corroboration of sequence data supports the good quality of our reference transcriptome. Moreover, proteomic data allowed the identification of up to 887 (PD) proteins for which no transcripts were observed, possibly due to a short half-life of the corresponding transcripts.

### Functional Annotation

All unique sequences were annotated using BLASTX based on sequence similarity searches against public NCBI non-redundant (nr) and UniProt databases [49], [50]. Among all sequences, 44 034 (36.1%; 53.0% of contigs/isotigs and 23.6% of singleton reads) had at least one significant hit to existing genes in the databases. This is nearly twice the number of annotated *Phalaenopsis* (only 22 234 transcripts; 51.9%) [35] and *Oncidium* sequences (22 810, or 44.8% of contigs; 23 591, or 19.6% of singleton reads) [34]. The remaining 77 883 sequences did not match any known sequences, and may be considered novel transcripts. Alternatively, these sequences may be too short to match known sequences in the databases, or they may be derived from untranslated or nonconserved regions with low homology to known protein sequences, as has been reported in several studies [51], [52], [53]. We observed that annotation success was positively correlated (R^2^ = 0.41, *p*<0.001; Fig. S2) with sequence length, similar to data shown by Hoffman [54]. Therefore, long transcripts without BLAST hits are most likely to represent novel genes in *Ophrys* (Fig. S2).

**Figure 4:**
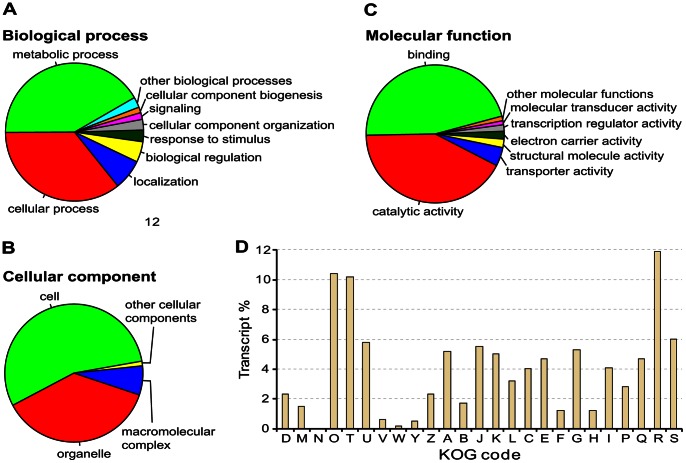
Functional annotation. *(A–C)* Pie charts showing the composition of 2^nd^ level GO terms of the *Ophrys* reference transcriptome, broken up into the three major GO categories: *(A)* biological process, *(B)* cellular components, and *(C)* molecular function. *(D)* Summary of KOG functional annotation of *Ophrys* transcripts. The KOG terms are: D: Cell cycle control, cell division, chromosome partitioning; M: Cell wall, membrane, envelope biogenesis; N: Cell motility; O: Posttranslational modification, protein turnover, chaperones; T: Signal transduction mechanisms; U: Intracellular trafficking, secretion, and vesicular transport; V: Defence mechanisms; W: Extracellular structures; Y: Nuclear structure; Z: Cytoskeleton; A: RNA processing and modification; B: Chromatin structure and dynamics; J: Translation, ribosomal structure and biogenesis; K: Transcription; L: Replication, recombination and repair; C: Energy production and conversion; E: Amino acid transport and metabolism; F: Nucleotide transport and metabolism; G: Carbohydrate transport and metabolism; H: Coenzyme transport and metabolism; I: Lipid transport and metabolism; P: Inorganic ion transport and metabolism; Q: Secondary metabolites biosynthesis, transport and catabolism; R: General function prediction only; S: Unknown function.

Possible functions of transcripts with significant BLAST hits were classified for the three main Gene Ontology (GO) functional categories: biological process, molecular function, and cellular component (GO level 2, Fig. 4; levels 1–12, Table S4). The largest number of transcripts (21 138 transcripts) was annotated by molecular function, followed by biological process (19 960) and cellular component (19 272). In the molecular function category, most transcripts were assigned to ‘binding’ (46.1%) and ‘catalytic activity’ (42.1%). Within biological process, the most abundant categories were ‘metabolic process’ (42.0%) and ‘cellular process’ (35.9%). For cellular components, ‘cell’ and ‘organelle’ had the highest number of transcripts (55.0% and 37.1%, respectively).

To obtain additional annotation information for *Ophrys* transcripts and to identify putative orthologues and paralogues, all sequences were compared to the eukaryotic clusters of orthologous groups of proteins (KOG) database [55]. In total, 24 412 transcripts (20.0%) were assigned to different eukaryotic orthologous groups (Fig. 4D). The two major functional groups assigned are ‘general function prediction only’ (2893 transcripts, 11.9%) and ‘posttranslational modification, protein turnover, chaperones’ (2527 transcripts, 10.4%).

### KEGG Pathways

The Kyoto Encyclopaedia of Genes and Genomes (KEGG) classification system provides an alternative functional annotation for genes according to their association with biochemical pathways [56]. To evaluate the completeness of the *Ophrys* reference transcriptome, transcripts were assigned to KEGG pathways and to enzyme commission (EC) numbers. A total of 7394 transcripts were assigned to KEGG pathways (Table 4) and the presence of *Ophrys* sequences for the majority of enzymes involved in essential biochemical pathways provides further evidence that the *Ophrys* transcriptome is relatively complete.

**Table 4:** Summary of KEGG pathway analysis.

KEGG pathways	KEGG sub-pathways	N transcripts	N enzymes
Metabolism	Amino Acid Metabolism	892	172
	Biosynthesis of Other Secondary Metabolites	252	37
	Carbohydrate Metabolism	1300	206
	Energy Metabolism	707	81
	Glycan Biosynthesis and Metabolism	173	35
	Lipid Metabolism	393	82
	Metabolism of Cofactors and Vitamins	444	63
	Metabolism of Other Amino Acids	195	35
	Metabolism of Terpenoids and Polyketides	87	28
	Nucleotide Metabolism	657	55
	Overview	1934	351
	Xenobiotics Biodegradation and Metabolism	170	30
Genetic Information Processing	Translation	84	20
Environmental Information Processing	Signal Transduction	66	9
Organismal Systems	Immune System	40	1

The number of *Ophrys* transcripts in a given sub-pathway, as well as the corresponding number of distinct enzymes in the KEGG database are shown.

### Protein Domains

A total of 20 858 unique *Ophrys* transcripts matched 4274 protein domains in the Pfam conserved domain database (Tables 5 and S5), with Pkinase, RRM_1 and PPR being the most highly represented classes. Among these, Pkinase represents a conserved protein domain containing the catalytic function of protein kinases, which play roles in various cellular processes including division, proliferation, apoptosis, and differentiation [57]. RRM proteins are the largest group of single strand RNA-binding proteins in eukaryotes that play important roles in RNA processing and protein synthesis regulation [58]. PPR repeat proteins represent the biggest multigene family in plants, and are involved in almost all stages of gene expression, including mRNA transcription, processing, splicing, editing, translation and stability [59]. Among the protein domains of the enzymes putatively involved in hydrocarbon and anthocyanin biosynthesis (Table 5), the AMP-binding and 2OG-FeII_Oxy domains were most highly represented.

**Table 5:** Summary of Pfam domains.

Accession	ID	Description	Occurrence
***Protein domains in the reference transcriptome***			
PF00069.19	Pkinase	Protein kinase domain	507
PF00076.16	RRM_1	RNA recognition motif. (a.k.a. RRM, RBD, or RNP domain)	237
PF01535.14	PPR	pentatricopeptide repeat	220
PF00400.26	WD40	WD domain, G-beta repeat	203
PF07714.1	Pkinase_Tyr	Protein tyrosine kinase	199
PF07727.8	RVT_2	Reverse transcriptase (RNA-dependent DNA polymerase)	190
PF00067.16	p450	Cytochrome P450	148
PF12854.1	PPR_1	Pentatricopeptide repeat	142
PF00665.20	rve	Integrase core domain	93
PF00004.23	AAA	ATPase family associated with various cellular activities (AAA)	90
PF00270.23	DEAD	DEAD/DEAH box helicase	90
PF00271.25	Helicase_C	Helicase conserved C-terminal domain	80
PF00153.21	Mito_carr	Mitochondrial carrier protein	77
PF00481.15	PP2C	Protein phosphatase 2C	75
PF00036.26	efhand	EF hand	74
	Other domains		18 055
***Protein domains in candidate genes for hydrocarbon biosynthesis***			
PF00501	AMP-binding	AMP-binding enzyme	62
PF01061	ABC2_membrane	ABC-2 type transporter	43
PF00106	adh_short	short chain dehydrogenase	35
PF00378	ECH	Enoyl-CoA hydratase/isomerase family	23
PF01553	Acyltransferase	Acyltransferase	13
PF03405.8	FA_desaturase_2	Fatty acid desaturase	13
PF08392.1	FAE1_CUT1_RppA	FAE1/Type III polyketide synthase-like protein	13
PF07993	NAD_binding_4	Male sterility protein	13
	Other domains		60
***Protein domains in candidate genes for anthocyanin biosynthesis***			
PF03171	2OG-FeII_Oxy	2OG-Fe(II) oxygenase superfamily	63
PF00201	UDPGT	UDP-glucoronosyl and UDP-glucosyl transferase	19
PF02797	Chal_sti_synt_C	Chalcone and stilbene synthases, C-terminal domain	10
PF00195	Chal_sti_synt_N	Chalcone and stilbene synthases, N-terminal domain	7
PF02431	Chalcone	Chalcone-flavanone isomerase	4

Highly abundant protein domains in the *Ophrys* reference transcriptome and among candidate genes for hydrocarbon and anthocyanin biosynthesis. ‘Occurrence’ lists the number of transcripts matching a given domain.

### Candidate Genes for Pollinator Attraction

#### Genes involved in VLCFA and hydrocarbon biosynthesis

**Figure 5:**
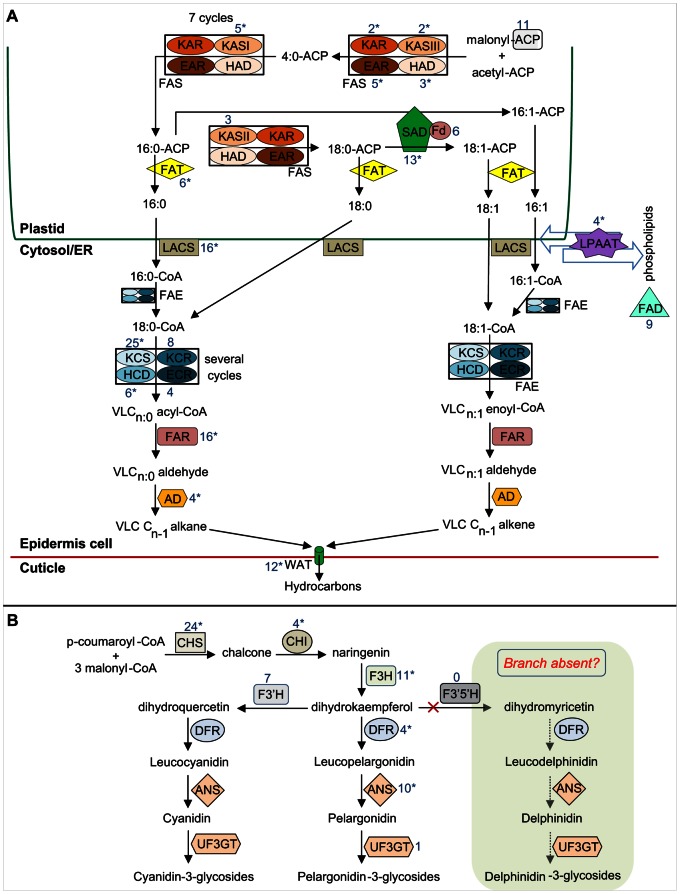
Candidate genes in biosynthetic pathways. Schematic diagrams of *(A)* hydrocarbon and *(B)* core anthocyanin biosynthesis, indicating candidate protein classes. The number of transcripts for a given candidate gene class is indicated in blue numbers, where an asterisk (*) indicates that a given candidate gene was confirmed at the protein level. Fatty acids in (*A*) are shown using C:D notation, where C is the number of carbon atoms and D is the number of double-bonds. Greyed, dotted arrows in panel *(B)* indicate metabolic reactions hypothesised to be absent. Protein abbreviations and further details on the listed candidates are provided in Table S6.

We combined transcriptome and proteome data sets to create a comprehensive resource for gene discovery in *Ophrys* orchids, with the aim of identifying candidate genes for pollinator attraction and reproductive isolation among *Ophrys* species. An important group of such candidate genes is putatively involved in hydrocarbon (and thus VLCFA) biosynthesis [17] (Fig. 5A). While all biosynthetic enzymes in the pathway are potential candidate genes for species-specific pollinator attraction, stearoyl-ACP desaturase (SAD) and β-ketoacyl-CoA synthase (KCS) enzymes are obvious *a priori* candidates for changes in hydrocarbon double-bonds and chain lengths, respectively [17], [60], which constitute the main odour differences among our study species. Homologues of all enzymes putatively involved in hydrocarbon biosynthesis were found in the *Ophrys* reference transcriptome, with 156 unique transcripts (132 putative genes; numbers include singleton reads) representing 20 candidate gene classes (Fig. 5A, Table S5). Seventy per cent (14/20) of these enzymes were confirmed by peptides (Table S6), including SAD and KCS. Proteins that were not confirmed by proteomics data were mostly membrane-associated (e.g., FAD or transporters) or very small (ferredoxin or ACP), and as such are less likely to be detected by shotgun proteomics.

Plant SAD proteins are a class of ubiquitous soluble enzymes that catalyse the insertion of a double-bond into acyl-ACP. Differential expression and/or activity of different orchid SADs is responsible for alkene double-bond differences among *O. sphegodes and O. exaltata* [20], [24]. Homologous to seven known *Arabidopsis SAD* genes [61], we identified 13 different *SAD* transcripts in 7 gene models (isogroups) from transcriptome assembly that likely correspond to 6 known *Ophrys SAD* genes [20], [24], [33]. This suggests that our knowledge of the identity of *Ophrys SAD* genes is relatively complete. KCS, a critical component of the fatty acid (FA) elongase (FAE) complex, catalyses the condensation of malonyl-CoA and fatty acyl-CoA to extend the FA by two carbon units [62]. KCS is a good candidate for the differences in alkene chain length between *O. sphegodes* and *O. garganica*. Twenty-one *KCS*-like genes have been annotated in the *Arabidopsis* genome [63], [64], [65], and we identified 25 *KCS* transcripts (24 gene models) in *Ophrys*. This high number of transcripts indicates ample potential for evolutionary change in *KCS-*like genes. However, experimental studies are required to test the role of *KCS* in changing hydrocarbon chain length in *Ophrys*.

#### Genes involved in anthocyanin biosynthesis

Anthocyanidin pigments are formed as part of the flavonoid pathway, accumulate in the vacuole of epidermal cells, and can be responsible for red, orange, purple, and blue colours in flowers [66], [67]. These pigments play important ecological functions, such as providing visual signals to attract pollinators from a distance. Because of their ubiquity in flowering plants, the biosynthesis and regulation of anthocyanins are well understood, and genes involved in these processes have been characterised in several orchid species (e.g. [68], [69]). While pollinator specificity in *Ophrys* is mostly due to hydrocarbon differences, floral coloration is involved in the mimicry of the pollinator female’s body colour [17]. For instance, *O. garganica* has darker flowers than *O. sphegodes* (see Fig. S1), corresponding to the darker body colour of its pollinator, the black *Andrena pilipes*. With one exception, homologues of all enzymes of the core anthocyanin biosynthesis pathway were found in the *Ophrys* reference transcriptome, with 61 unique transcripts (59 putative genes), representing 7 candidate enzyme classes (Fig. 5B, Table S6). Seventy-one per cent (5/7) of these enzymes were confirmed by peptides (Table S6). Interestingly, the one exception was flavonoid 3′,5′-hydroxylase (F3′5′H), for which no transcripts (or peptides) were found. It is possible that this absence of *F3*′*5*′*H* is not due to limited transcriptome coverage, but reflects the biology of (rather reddish) *Ophrys* flowers, because F3′5′H is required for the formation of (often bluish) delphinidin pigments [66], [70]. Similar situations are known in several other plants without delphinidin pigments (e.g., *Ipomoea, Rosa, Dianthus,* and *Chrysanthemum*) [71], in which *F3*′*5*′*H* either is not expressed or was lost from the genome (e.g. in *Arabidopsis thaliana*) [72]. Given that delphinidin pigments may be present in the distantly related *O. speculum* [73], this loss of *F3*′*5*′*H* transcript and/or gene may only have occurred relatively recently.

#### Transcription factors

**Figure 6:**
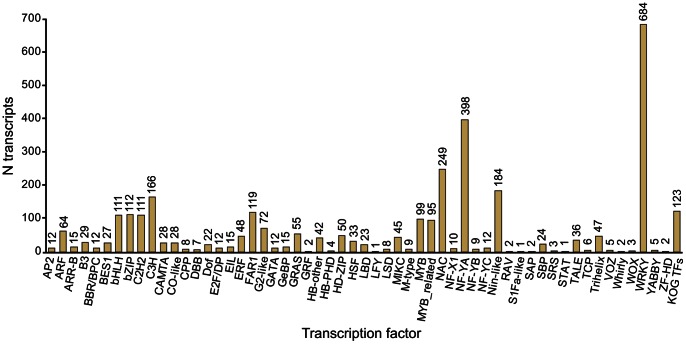
Transcription factors. Bar plot showing the number of transcripts in different transcription factor classes; additional TFs identified by KOG annotation are plotted as a separate column.

Transcription factors (TFs) are important regulators of gene expression in response to plant developmental processes and environmental factors [74]. Since they are potential candidates for species differences in pollinator attraction, TFs in the *Ophrys* transcriptome were identified by a comparison to *Arabidopsis thaliana* and *Oryza sativa* transcription factor databases [75] and by KOG annotation. Overall, 3319 unique transcripts (2.7% of the *Ophrys* transcriptome), encoding members of 56 putative TF families, were identified (Fig. 6; Table S7), which is higher than the number of TFs identified in *Phalaenopsis* (786 transcripts, 1.83%). The most abundant TF families were WRKY, NF-YA and NAC factors (Fig. 6). Moreover, LFY, M-type, STAT, VOZ and WOX factors, which had not been found in the *Phalaenopsis* transcriptome [35], were detected in the *Ophrys* transcriptome. Other abundantly represented TF families in our dataset include Myb, bHLH and MADS factors.

MADS-domain (M-type and MIKC) TFs are involved in controlling all major aspects of plant development [76] and have been shown to regulate anthocyanin biosynthesis in pigmented tubers of sweet potato [77]. MADS-domain proteins are important regulators of floral development [78], [79], and duplications of B-class MADS-box genes have been implicated in the evolution of complex orchid flowers [80]. For instance, the combinatorial expression of several paralogues is thought to specify flower labellum identity, which may also depend on the action of TCP TFs [80]. In the *Ophrys* transcriptome, MADS-domain factors were represented by 56 transcripts. These included 4 transcripts of *GLOBOSA*/*PISTILLATA* and 3 transcripts of *DEFICIENS*/*APETALA3* (clade 2, 3 and 4 [80], [81]; Table S8) B-class gene homologues. Moreover, 6 TCP TFs were found in the transcriptome. These genes represent candidate genes for labellum development.

The Myb and basic helix-loop-helix (bHLH) families are the largest and second largest classes of TFs in plants, respectively [82]. In rice and *Arabidopsis* there are 339 and 230 Myb factors, and 162 and 111 bHLH TFs, respectively [82]. Myb (Myb and MYB-related) factors account for 197 transcripts in the *Ophrys* transcriptome, whereas the bHLH family is represented by 111 transcripts. Myb TFs are especially interesting candidate genes, because they are implicated in a range of functions, including the regulation of secondary metabolism [83], [84], [85], [86], cell morphogenesis [87], [88], [89], control of the cell cycle [90], [91], floral and seed development [92], [93], [94], [95], responses to biotic and abiotic stresses [96], [97], [98], [99], and light and hormone signalling pathways [100], [101], [102]. Particularly, Myb factors controlling cell shape as well as VLCFA and anthocyanin biosynthesis may be important candidates for changes in floral traits like odour, colour and epidermal cell shape, all of which are involved in specific pollinator attraction in *Ophrys* [17].

One interesting Myb gene, *MYB30*, has been found to be involved in VLCFA biosynthesis by controlling the expression of four enzymes forming the FAE complex in *Arabidopsis* [103]. Two transcripts homologous to *MYB30* were identified in *Ophrys* (Table S7). A specific group of Myb factors, R2R3 MYBs, interacts with bHLH factors and WD-repeat (WDR) proteins to form the MYB–bHLH–WDR (MBW) complexes that regulate anthocyanin biosynthesis [70]; R2R3 MYB subgroup 6 (SG6) TFs such as *AtMYB113*, *AtMYB114*, *AtMYB75/PAP1* and *AtMYB90/PAP2* activate anthocyanin biosynthesis in many different species [83], [104], [105]. Four transcripts represent SG6 genes in the *Ophrys* transcriptome (Table S7).

Conical cells may enhance the colour intensity and brightness of petal surface and, thereby, increase flower attractiveness to the pollinators [106], [107], [108], [109]. *MIXTA*, a Myb-related TF, drives the formation of conical epidermal cells from the flat epidermal cells of the snapdragon, *Antirrhinum majus* [109], [110], and *AmMYBML1* encodes a similar R2R3 MYB TF that has a role in controlling trichome, conical cell and mesophyll cell morphogenesis in the ventral petal of *Antirrhinum* flowers [111]. One *AmMYBML1*-like and three *MIXTA*-like genes were identified in *Ophrys* (Table S7).

Taken together, our *Ophrys* reference transcriptome with its focus on flowers allowed us to identify several putative transcription factors that may be relevant to our understanding of the molecular basis of pollinator attraction. This includes MADS, TCP and Myb factors that may be involved in the orchid’s mimicry of pollinator females by regulating odour, colour and floral morphological traits. Our data thus provide a starting point for investigating the molecular mechanisms of pollinator attraction and adaptation.

### Evolutionary Implications

#### Evolution of sexual deception

Sexual deception in *Ophrys* may have evolved from food deception [112], and alkene production (and possibly labellum size) may have served as pre-adaptations for this mode of pollination [112], [113]. Floral morphological development, hydrocarbon and anthocyanin biosynthesis play important roles in facilitating sexually deceptive pollination [17]. These pathways, however, are likely to be similar in *Ophrys* and related genera, which share many of its floral features and (pre-)adaptive traits, although with quantitative differences (e.g. in alkene levels [113]). This implies that sexual deception likely arose by modification of existing pathways, possibly by regulatory changes or changes associated with gene duplication (and subsequent neo- and subfunctionalisation). Therefore, comparative genomic studies incorporating other orchid species may lead to the identification of the genetic changes that allowed the evolution of sexual deception.

#### Molecular mechanisms of reproductive isolation

All biosynthetic and regulatory candidate genes uncovered in the transcriptome were present in all species, with the exception of *F3*′*H* and some transcription factors, all of which were only detected in *O. sphegodes* (Tables S6 and S7). However, this may be due to lower sequence coverage in the other two species, which might have prevented detection of rare transcripts. The widespread presence of candidate gene transcripts in all species therefore suggests that species differences in pollinator attraction, and consequently reproductive isolation, are unlikely to be caused by the simple presence or absence of expression in any candidate gene class. Rather, it may be expected that subtler changes, such as paralogue-, isoform-, or allele-specific sequence and/or expression changes, or even epigenetic phenomena [114], [115], underlie reproductive isolation. This holds true at least for *SAD* genes specifying alkene double-bond positions [20], [24] and appears similarly plausible for *KCS* and hydrocarbon chain length, where the presence of 25 transcript sequences presages complexity. More detailed studies that tease apart the effects of different gene copies, alleles and quantitative expression changes are required to understand the exact molecular architecture of pollinator-mediated reproductive isolation among *Ophrys* species.

### Conclusions

Here, we employed high throughput next-generation sequencing technologies combined with shotgun proteomics to provide the first floral reference transcriptome and proteome data for sexually deceptive *Ophrys* from the Orchidoideae subfamily of orchids, for which proteome data were previously absent and sequence information scarce. We thereby provide significant resources for gene discovery and systems biology in orchids in general, by enabling sequence comparisons among disparate lineages of orchids. Likewise, our data set considerably expands the resources available for sexually deceptive plants, and provides an opportunity to advance our understanding of the molecular basis of plant-pollinator interactions, as well as pollinator-mediated selection and speciation. Our data are relevant for the characterisation of candidate genes for these and other processes, as demonstrated here by the identification of genes potentially involved in plant pseudo-pheromone biosynthesis and regulation. Based upon *a priori* knowledge and the abundance of these gene classes in the *Ophrys* transcriptome, especially *KCS* biosynthetic genes and *Myb* transcription factors warrant further attention as candidate genes for differences in specific pollinator attraction. Subtle changes in such genes may be responsible for reproductive isolation among sexually deceptive orchids.

## Materials and Methods

### Plant Materials

Plant material of *O. exaltata* subsp. *archipelagi* (Gölz & Reinhard) Del Prete, *O. garganica* Nelson, and *O. sphegodes* Miller for 454 sequencing was grown in a greenhouse at the Botanic Garden of the University of Zurich, Switzerland. Additional flower labellum samples of these three species were used for proteome analysis and two further *O. sphegodes* flower labella for EST library and Illumina Solexa sequencing, all of these samples being from different plants. All flowers used in this study were unpollinated. Plant tissues were collected, flash-frozen in liquid nitrogen and stored at −80°C until RNA or protein extraction. For 454 sequencing, the following tissues were used: leaves, bracts, labella, sepals, petals and columns, both from open flowers and flower buds from all developmental stages available on the sampled plants, the smallest available bud being five positions from the latest open flower on the inflorescence.

### cDNA Normalisation and 454 Sequencing

Total RNA was extracted separately from different tissues (see above) collected from three different individual plants per species (21 RNA samples/species) using TRIzol® reagent (Invitrogen) and the supplier’s protocol. Extracts were further purified by using RNeasy MinElute Cleanup columns (Qiagen) according to the manufacturer’s protocol. The extracted RNAs were analysed for potential degradation by gel electrophoresis and on a Bioanalyzer 2100 (Agilent), and RNA concentration was quantified using the fluorometric Qubit Quantitation Platform (Invitrogen). Equal amounts of RNA from each biological individual were pooled to yield one RNA sample for each *Ophrys* species. To avoid genomic DNA contamination, RNA was treated with RNase-free DNase I (Qiagen). Full-length double-stranded cDNA was synthesised from 0.5 µg pooled total RNA using SMARTer PCR cDNA Synthesis Kit (Clontech, Palo Alto, CA, USA) according to the manufacturer’s instructions. To enhance gene discovery, the contribution of highly abundant transcripts was reduced before sequencing. To do so, 1 µg of each cDNA was normalised using the Trimmer cDNA Normalization Kit (Evrogen, Moscow, Russia), according to the manufacturer’s instructions.

Approximately 500 ng of normalised cDNA of each sample were used to generate a single strand cDNA transcriptome library for the Roche/454 Life Sciences GS-FLX Titanium platform (Roche, Basel, Switzerland) following the Rapid Library Preparation Method Manual. Briefly, cDNA of each sample was sheared by nebulisation to produce fragments of approximately 300–400 bp, and oligonucleotide adapters were ligated to the fragmented cDNA. One adapter contained a barcode sequence that was used to discriminate the samples (i.e. species) from each other after sequencing, as all libraries were combined in a single pool. Transcriptome library sequencing was then performed according to the Roche GS-FLX XLR70 Titanium emPCR and sequencing manuals. The pooled sample was sequenced on a full picotitre plate on a Genome Sequencer FLX Instrument, according to the manufacturer’s instructions, at the Functional Genomics Centre Zurich, Switzerland.

### EST Library Preparation and Sequencing

Poly (A)^+^ RNA was purified from 2 µg *O. sphegodes* flower labellum total RNA using the Oligotex mRNA isolation kit (Qiagen AG, Hombrechtikon, Switzerland) according to the manufacturer’s instructions. One standard cDNA library was prepared using the Creator SMART cDNA Library Construction Kit and the protocol provided, except that insert size selection was performed using Zymoclean Gel DNA Recovery Kit (Zymo Research, Orange, CA, USA). Oligo-dT-primed cDNA inserts larger than 1 kb were directionally cloned in pDNR-LIB vector (Clontech, Palo Alto, CA, USA) and transformed into XL-10 Gold Kan^+^ ultracompetent *Escherichia coli* (Stratagene, LaJolla, CA, USA). Colony PCR reactions were performed to test the library efficiency and insert size range. The library was stored by the addition of glycerol (20% v/v final concentration) and sent for Sanger sequencing (Applied Biosystems) at the Purdue University sequencing platform, West Lafayette, Indiana (USA).

### Illumina Solexa Sequencing

Total RNA of one *O. sphegodes* flower labellum was used for an RNA-Seq experiment by Illumina Solexa sequencing at BGI Shenzhen (China), following Illumina’s sample preparation guidelines. Briefly, poly(A)^+^ was purified from total RNA and fragmented, cDNA synthesised and adapters ligated. After size selection, cDNA was subjected to Solexa paired-end sequencing on an Illumina Genome Analyzer II generating 75 nt long reads.

### Processing of Sequence Reads

For Sanger reads, base calling, masking of vector sequences and low quality ends were done with phred (version 071220) via the phredPhrap script (version 080818) in consed (version 20.0) [116], and trimming of poly-A/T tails with seqclean (http://compbio.dfci.harvard.edu/tgi/software/). Raw Solexa reads were filtered by quality using the manufacturer’s software and default parameters. Raw 454 sequencing data was obtained with the GS Run Processor 2.5.3 (Roche) using shotgun quality filtering and trimming as defined by the default settings. Raw 454 reads were first trimmed of adapter sequences used in cDNA library preparation and normalisation with newbler in the Roche 454 Software Suite 2.5.3 (454 Life Science, Branford, CT). MEGABLAST 2.2.21 [117] was run to ascertain that trimmed reads were clean of any adapter and primer sequences. Trimmed 454 reads were further processed with seqclean to remove low complexity and low quality reads, and to remove any left-over poly-A/T tails.

### Transcriptome Assembly

*O. sphegodes* Solexa reads were first assembled into contigs using SOAPdenovo [118], using paired-end information to link contigs into scaffolds, and where possible, to fill gaps with reads. Scaffolds were clustered using TGICL [119], each cluster producing one or more consensus sequences; unclustered scaffolds were termed singleton contigs. *O. sphegodes* Sanger reads and 454 reads from all three species were both (1) assembled separately for each species and (2) pooled and assembled with 454 newbler 2.5.3 (454 Life Science, Branford, CT). The pooled assembly was further merged with the *O. sphegodes* Solexa assembly using minimus2 under the criteria of minimum 40 bases overlap with at least 94% identity [120]. The merged assembly from Sanger, 454 and Solexa data is referred to as the *Ophrys* reference transcriptome.

### Transcriptome Annotation and Analysis

Assembled sequences and singleton reads were compared to the NCBI non-redundant (nr) database using BLASTX [121]. Based on the search results, Gene Ontology (GO) term annotation was performed to predict the function of the sequences using BLAST2GO software [122]. Enzyme commission (EC) number and Kyoto Encyclopaedia of Genes and Genomes (KEGG) pathway [56], [123] were inferred from GO annotations using the same software. Moreover, GO, EC and KEGG annotation was also done with annot8r based on BLASTX results from searches against the UniProt database. Orthologous genes (KOG annotation) were identified by searching against the NCBI KOG database using RPSTBLASTN 2.2.26 [121]. Coding regions were predicted using ESTScan 3.0.3 [124] and then compared to the Pfam protein domain database [125] using pfam_scan 1.3 [126].

### Read Mapping

Read mapping was performed for purposes of data set cross-validation. Solexa reads were mapped onto 454/Sanger singleton reads using Bowtie 0.12.7 [127] (parameters: ‘-n 1 -l 35 -e 100 -m 5–best –strata’) as independent evidence for singleton reads. 454 reads were mapped back on the reference transcriptome using the BWA-SW algorithm in BWA 0.5.9 [128] (with parameter ‘-s 5′).

### Identification of Candidate Genes

*A priori* candidate genes were identified by homology to genes of known or putative function in model organisms. To identify hydrocarbon and anthocyanin biosynthetic genes, a BLASTN search of the *Ophrys* reference transcriptome against *Arabidopsis thaliana* TAIR10 coding sequences was performed (at e-value of<E-03), retaining information of the best BLAST hit for each orchid transcript. Homologues of *Arabidopsis* genes for selected gene classes were considered candidate genes. In addition, text/term searches of BLAST/NR best hits, GO annotations and EC numbers were used to identify candidate orchid genes. For transcription factor identification, KOG searches for transcription terms were performed alongside BLAST searches against *A. thaliana* and *Oryza sativa* TF databases publically available from PlantTFDB 2.0 (which currently does not contain sequences from Asparagales) [75]. TFs of special interest, putatively involved in the regulation of VLCFA, anthocyanin biosynthesis and flower morphology, were identified from the literature.

### Shotgun Proteomics

Frozen labellum tissue of an unpollinated flower from each study species was ground to powder without allowing it to thaw, and resuspended in 150 µL urea protein extraction buffer (65 mM Tris-HCl, 8 M urea, 10% glycerol, 5% β-mercaptoethanol, 2% SDS, 0.025% bromophenol blue), denatured and separated by SDS-PAGE, sliced (13 gel slices per sample) and trypsinised as described previously [129]. Proteins were subjected to electrospray ionisation-based LC-MS/MS analysis with a 2D linear ion trap Finnigan LTQ (Thermo Electron Corporation), equipped with an Ultimate Nano HPLC System (Dionex Corporation) exactly as described by Grobei et al. [129].

### Proteome Analysis

MS/MS-derived spectra were searched against different peptide databases using Mascot Search Engine version 2.3 (Matrix Science Ltd., UK). The databases used were SwissProt and TAIR9 [130], [131], including decoys and known contaminants (as in Grobei et al. 2009 [129]), and (1) orchid peptides predicted by ESTScan and (2) a 6-frame translation of the orchid transcriptome. Mascot searches, with a peptide mass tolerance of 3 Da, allowed for one trypsin miscleavage, for Met oxidation and Cys carbamidomethylation, and were further analysed and validated in R 2.14.2 [132] and Scaffold 3.3 (Proteome Software Inc., USA), which uses the Peptide-Prophet and Protein-Prophet algorithms [133], [134]. Spectra from known contaminants were removed from the data set for final analysis.

## Supporting Information

Figure S1 **Comparison of Ophrys flowers.** Five flowers each from different individuals of *(A) O. exaltata* subsp. *archipelagi*, *(B) O. sphegodes* and *(C) O. garganica*, showing inter-species and intra-species variation (plants all from Gargano, Southern Italy). Images were scaled for comparison, the white bar indicating 1 cm. *O. sphgodes* tends to have comparatively small flowers with a brown labellum and a greenish perigon, whereas *O. garganica* flowers are usually larger, with a darker labellum and sometimes coloured petals. *O. exaltata* tends to have comparatively large flowers with a slightly elongated brown labellum, typically with a protrusion at its apex, and usually a white perigon. The speculum (brighter, more reflective part of the labellum) can be quite variable in all species, and they all have longer trichomes at the sides of the labellum (‘hairy margin’) as compared to its centre. Micromorphological features of *Ophrys* flowers are described elsewhere [15].
(PDF)

Figure S2 **Transcript length/annotation relationship.** Plot showing the percentage of hits with annotation information from NCBI nr and UniProt databases versus sequence length.
(PDF)

Table S1 **Summary of sequencing data.** This table lists details of the 454, Sanger and Solexa sequence data sets obtained in this study.
(DOCX)

Table S2 **List of orchid proteins.** This table lists all proteins matching sequences in the *Ophrys* transcriptome by their transcriptome sequence ID. In the column HQ, an asterisk (*) denotes proteins in the HQ data set (no asterisk indicates PD data set). The ‘Species’ column indicates in which orchid species a given protein was found, where: E, *O. exaltata*; G, *O. garganica*; S, *O. sphegodes*. In the column ‘Orchid-only’, y/n (yes/no) indicate if a given protein was found only in the *Ophrys* transcriptome (y) or if it also matched proteins in the TAIR and/or SwissProt databases (n). The ‘Description’ column refers to the term provided in the transcript ID’s best BLASTX/nr database hit.
(XLSX)

Table S3 **List of proteins without transcript.** This table lists all proteins that were identified in the SwissProt/TAIR9 databases, but did not match any sequence in the *Ophrys* transcriptome, listing the protein description, source database and associated accession numbers. In the column HQ, an asterisk (*) denotes proteins in the HQ data set (no asterisk indicates PD data set). The ‘Species’ column indicates in which orchid species a given protein was found, where: E, *O. exaltata*; G, *O. garganica*; S, *O. sphegodes*.
(XLSX)

Table S4 **GO classification.** This table contains the GO classification of the assembled sequences into three main categories (biological function, cellular component and molecular function) at all levels (1–12). ‘All species’ lists the number of transcripts in a given category in the *Ophrys* reference transcriptome, and additional columns list the corresponding number of transcripts for the individual species separately.
(XLS)

Table S5 **Pfam protein domains.** This table lists the Pfam annotations in the *Ophrys* reference transcriptome by transcript ID.
(XLSX)

Table S6 **List of candidate genes.** Details of transcripts encoding candidate biosynthetic proteins putatively involved in hydrocarbon and anthocyanin biosynthesis, sorted alphabetically per category. TAIR ID lists the *Arabidopsis* gene for which homologues were found; EC is the enzyme commission number searched for a given candidate protein; ‘N transcripts’ lists the number of unique transcripts matching a candidate protein category in the *Ophrys* reference transcriptome (counting singleton reads as transcripts); ‘N gene models’ is composed of the number of isogroups from sequence assembly plus transcripts that were not assigned to isogroups (so that for instance, singleton reads would be counted as a new gene model); ‘Unique transcripts’ lists the unique transcriptome sequence IDs. Species with transcripts and peptides list in which *Ophrys* species transcripts or peptides were found, respectively, where: E, *O. exaltata*; G, *O. garganica*; S, *O. sphegodes*. In the column ‘Species with peptides’, an asterisk indicates that a protein was part of the HQ data set (no asterisk, PD data set). The ordering of elements in the two rightmost columns corresponds to the ordering in the ‘Unique transcripts’ column.
(DOCX)

Table S7 **List of transcription factors.** This table lists the identification method (TFDB, transcription factor database; KOG, KOG annotation) for TF identification, along with the unique transcript ID and annotation details (accession number or KOG term). The column ‘Transcription Factor’ lists the TF class identified from the database or the KOG description term, as appropriate. Species with transcripts or peptides for a given TF are listed, where: E, *O. exaltata*; G, *O. garganica*; S, *O. sphegodes*. Proteins identified in the HQ data set are marked with an asterisk. The Comments column provides further information on TFs of special interest. In addition to the TFs listed in this table, one additional transcript (transcript ID 3673) was found to have significant BLAST homology (e-value 6E–12) to a TF of interest, namely *Antirrhinum majus AmMYBML1*-like (AY661653.1).
(XLSX)

Table S8 **BLAST results for DEF/AP3 and GLO/PI MADS-box gene homologues.** Table of BLASTN results, showing top 10 hits against the NCBI nr database (sorted by e-value) for each putative *Ophrys* B-class MADS-box gene homologue identified in the *Ophrys* reference transcriptome. The column ‘Lineage’ lists the gene lineage (*DEF* clades 1, 2, 3 and 4; Orchidoideae *GLO1* and *GLO2*) assigned to the accession number of a BLAST hit, as defined by Mondragón-Palomino & Theißen (2008; [81]) for *DEF*, and Kim & al. (2007; [135]) and Cantone & al. (2011; [136]) for *GLO*. The best lineage assignment is highlighted in bold for each transcript. Accession numbers with defined lineage are as follows: *DEF* clade 1: AY378149, DQ119838, DQ683575, FJ804097, FJ804106, FJ804115; clade 2: AY196350, AY378148, FJ804098, FJ804105, FJ804111; clade 3: AY378150, AB232663, DQ119839, FJ804099, FJ804107, FJ804110, FJ804117; clade 4: AY378147, FJ804108, FJ804112, FJ804116; *GLO1*: AB232665, AB450305, AB450307, AB450310, AB450302, AB450308, AB450309, AB450299, AB450303, AB450306; *GLO2*: AB232664, AB537512, AB537507, AB537511, AB537513, AB537509, AB537508, AB537506, AB537504, AB537510.
(XLSX)

File S1 **This zip-compressed file contains (1) the assembled Ophrys reference transcriptome (FASTA), along with (2) an MD5 check-sum, (3) an FAI index file, (4) 454 isotig to isogroup (transcript to gene) mapping (tab-delimited text), and (5) a text file describing the naming scheme for the transcript identifiers used.**
(ZIP)

File S2 **This spreadsheet document contains the annotation information for the** ***Ophrys*** **reference transcriptome in several tables.**
(XLSX)
