# Supplementary figures and images for: Transcriptome and Proteome Data Reveal Candidate Genes for Pollinator Attraction in Sexually Deceptive Orchids
Source: PLoS One. 2013 May 29;8(5):e64621. doi: 10.1371/journal.pone.0064621 (PMC3667177; doi:10.1371/journal.pone.0064621)

**A**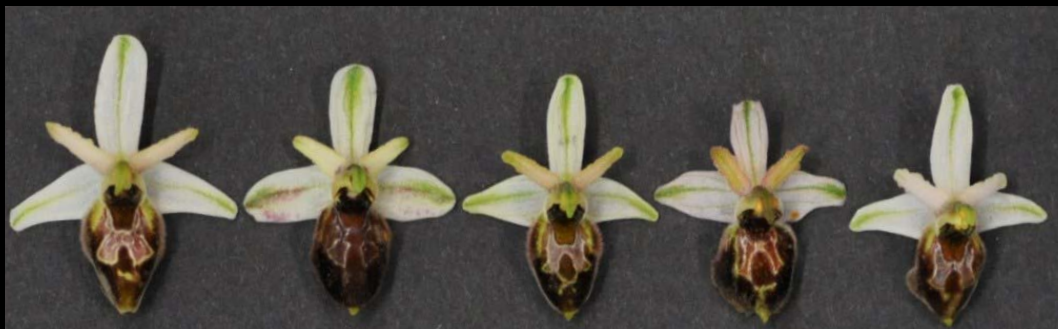**B**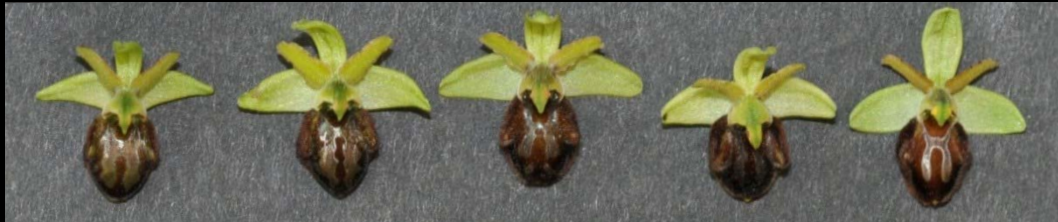

1 cm

**C**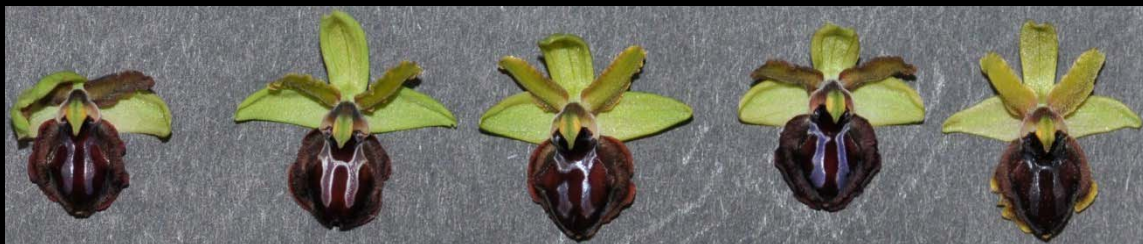

Supplement: Figure S1 — Comparison of Ophrys flowers. Five flowers each from different individuals of (A) O. exaltata subsp. archipelagi, (B) O. sphegodes and (C) O. garganica, showing inter-species and intra-species variation (plants all from Gargano, Southern Italy). Images were scaled for comparison, the white bar indicating 1 cm. O. sphgodes tends to have comparatively small flowers with a brown labellum and a greenish perigon, whereas O. garganica flowers are usually larger, with a darker labellum and sometimes coloured petals. O. exaltata tends to have comparatively large flowers with a slightly elongated brown labellum, typically with a protrusion at its apex, and usually a white perigon. The speculum (brighter, more reflective part of the labellum) can be quite variable in all species, and they all have longer trichomes at the sides of the labellum (‘hairy margin’) as compared to its centre. Micromorphological features of Ophrys flowers are described elsewhere [15]. (PDF) [file pone.0064621.s001.pdf]

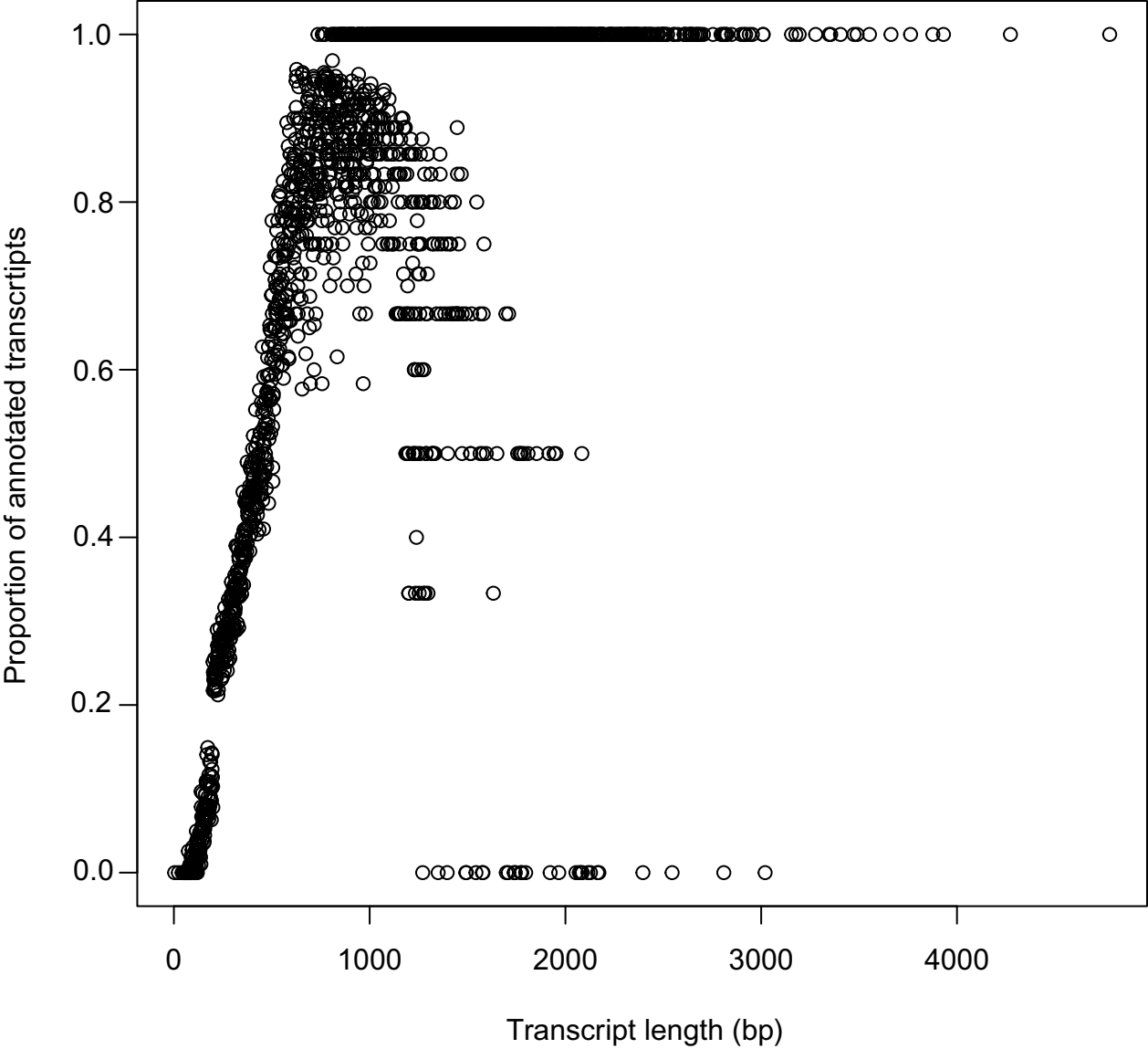

Supplement: Figure S2 — Transcript length/annotation relationship. Plot showing the percentage of hits with annotation information from NCBI nr and UniProt databases versus sequence length. (PDF) [file pone.0064621.s002.pdf]
