# Supplementary material for: Transcriptome and Proteome Data Reveal Candidate Genes for Pollinator Attraction in Sexually Deceptive Orchids
Source: PLoS One. 2013 May 29;8(5):e64621. doi: 10.1371/journal.pone.0064621 (PMC3667177; doi:10.1371/journal.pone.0064621)
Supplement: Table S1 — Summary of sequencing data. This table lists details of the 454, Sanger and Solexa sequence data sets obtained in this study. (DOCX) [file pone.0064621.s003.docx]

***Table S1. Summary of sequencing data.*** This table lists details of the 454, Sanger and Solexa sequence data sets obtained in this study.

| **Data** | ***O. exaltata*** | ***O. garganica*** | ***O. sphegodes*** | **Total** |
| --- | --- | --- | --- | --- |
| *454 data* (raw/processed) | | | | |
| 454 reads | 151 599 /  150 735 | 217 756 /  216 621 | 159 728 /  158 628 | 529 083 / 525 984 |
| Sequenced bases (nt) | 27 768 258 / 16 578 183 | 43 054 803 / 28 030 266 | 34 397 732 / 26 733 760 | 105 220 793 /  71 342 209 |
| Average read length^1^ (nt) | 200 | 225 | 229 | 218 |
| *Sanger data* (raw/clean^2^) | | | | |
| Sanger reads | – | – | 1920 / 1915 | 1920 / 1915 |
| Sequenced bases (nt) | – | – | 2 693 005 / 1 688 741 | 2 693 005 / 1 688 741 |
| Average read length (nt) | – | – | 1403 / 882 | 1403 / 882 |
| *Illumina Solexa data* | | | | |
| Paired-end reads^3^ | – | – | 16 751 644 | 16 751 644 |
| Sequenced bases (nt) | – | – | 2 512 746 600 | 2 512 746 600 |

^1^After quality filters.

^2^Bases that passed the masking of vector sequences and removal of low-quality ends.

^3^Reads were 75 nt long.
