# Supplementary material for: Transcriptome and Proteome Data Reveal Candidate Genes for Pollinator Attraction in Sexually Deceptive Orchids
Source: PLoS One. 2013 May 29;8(5):e64621. doi: 10.1371/journal.pone.0064621 (PMC3667177; doi:10.1371/journal.pone.0064621)
Supplement: Table S6 — List of candidate genes. Details of transcripts encoding candidate biosynthetic proteins putatively involved in hydrocarbon and anthocyanin biosynthesis, sorted alphabetically per category. TAIR ID lists the Arabidopsis gene for which homologues were found; EC is the enzyme commission number searched for a given candidate protein; ‘N transcripts’ lists the number of unique transcripts matching a candidate protein category in the Ophrys reference transcriptome (counting singleton reads as transcripts); ‘N gene models’ is composed of the number of isogroups from sequence assembly plus transcripts that were not assigned to isogroups (so that for instance, singleton reads would be counted as a new gene model); ‘Unique transcripts’ lists the unique transcriptome sequence IDs. Species with transcripts and peptides list in which Ophrys species transcripts or peptides were found, respectively, where: E, O. exaltata; G, O. garganica; S, O. sphegodes. In the column ‘Species with peptides’, an asterisk indicates that a protein was part of the HQ data set (no asterisk, PD data set). The ordering of elements in the two rightmost columns corresponds to the ordering in the ‘Unique transcripts’ column. (DOCX) [file pone.0064621.s008.docx]

***Table S6. List of candidate genes.*** Details of transcripts encoding candidate biosynthetic proteins putatively involved in hydrocarbon and anthocyanin biosynthesis, sorted alphabetically per category. TAIR ID lists the *Arabidopsis* gene for which homologues were found; EC is the enzyme commission number searched for a given candidate protein; ‘N transcripts’ lists the number of unique transcripts matching a candidate protein category in the *Ophrys* reference transcriptome (counting singleton reads as transcrips); ‘N gene models’ is composed of the number of isogroups from sequence assembly plus transcripts that were not assigned to isogroups (so that for instance, singleton reads would be counted as a new gene model); ‘Unique transcripts’ lists the unique transcriptome sequence IDs. Species with transcripts and peptides list in which *Ophrys* species transcripts or peptides were found, respectively, where: E, *O. exaltata*; G, *O. garganica*; S, *O. sphegodes*. In the column ‘Species with peptides’, an asterisk indicates that a protein was part of the HQ data set (no asterisk, PD data set). The ordering of elements in the two rightmost columns corresponds to the ordering in the ‘Unique transcripts’ column.

| **Candidate enzyme/protein** | **TAIR ID** | **EC** | **N transcripts** | **N gene models** | **Unique transcripts** | **Species with transcripts** | **Species with peptides** |
| --- | --- | --- | --- | --- | --- | --- | --- |
| ***Hydrocarbon biosynthesis*** | | | | | | | |
| acyl carrier protein (ACP) | AT1G65290 |  | 11 | 6 | 2201 | E, G, S |  |
|  | AT2G44620 |  |  |  | 2461 | E, G, S |  |
|  | AT3G05020 |  |  |  | 2660 | E, G, S |  |
|  | AT4G25050 |  |  |  | newbler-isotig00220 | E, G, S |  |
|  | AT5G27200 |  |  |  | newbler-isotig00221 | E, G, S |  |
|  |  |  |  |  | newbler-isotig00222 | E, G |  |
|  |  |  |  |  | newbler-isotig00223 | E, G, S |  |
|  |  |  |  |  | newbler-isotig00224 | E, G, S |  |
|  |  |  |  |  | newbler-isotig00225 | E, G, S |  |
|  |  |  |  |  | newbler-isotig02318 | E, G, S |  |
|  |  |  |  |  | Singletons218576 | S |  |
| acyl-ACP thioesterase (FAT ) | AT1G08510 | 3.1.2.14 | 6 | 6 | 1135 | E, G, S | E, G, S* |
|  | AT3G25110 |  |  |  | 3012 | E, G, S |  |
|  |  |  |  |  | Singletons13442 | S |  |
|  |  |  |  |  | Singletons216692 | G, S |  |
|  |  |  |  |  | Singletons220297 | S |  |
|  |  |  |  |  | Singletons222752 | G |  |
| aldehyde decarbonylase (AD) | AT1G02190 | 4.1.99.5 | 4 | 1 | newbler-isotig00185 | E, G, S | E, G, S* |
|  | AT1G02205 |  |  |  | newbler-isotig00186 | E, G, S | E, G, S* |
|  | AT2G37700 |  |  |  | newbler-isotig00187 | E, G, S |  |
|  | AT5G28280 |  |  |  | newbler-isotig00188 | E, G, S |  |
| β-hydroxyacyl-ACP dehydratase (HAD) | AT2G22230 | 4.2.1.60 | 3 | 3 | 1353 | E, G, S | E, G, S* |
|  |  |  |  |  | Singletons212081 | S |  |
|  |  |  |  |  | Singletons227086 | S |  |
| β-hydroxyacyl-CoA dehydratase (HCD) | AT5G10480 | 4.2.1.17 | 6 | 6 | 902 | E, G, S | S* |
|  |  |  |  |  | GZ44Y0R01B9Z68^1^ | G |  |
|  |  |  |  |  | GZ44Y0R01CERPY^1^ | G |  |
|  |  |  |  |  | GZ44Y0R01DGN5T^1^ | G |  |
|  |  |  |  |  | Singletons218808 | G | E, G, S* |
|  |  |  |  |  | Singletons225098^1^ | S |  |
| β-ketoacyl-ACP reductase (KAR) | AT1G24360 | 1.1.1.100 | 2 | 2 | 934 | E, G, S | E, G, S* |
|  | AT3G55310 |  |  |  | 956 | E, G, S | E, G, S* |
| β-ketoacyl-CoA reductase (KCR) | AT1G67730 | 1.1.1.35 | 8 | 8 | 4186 | E, G, S |  |
|  |  |  |  |  | 5351 | E, G, S |  |
|  |  |  |  |  | GZ44Y0R01B9Z68^1^ | G |  |
|  |  |  |  |  | GZ44Y0R01CERPY^1^ | G |  |
|  |  |  |  |  | GZ44Y0R01DGN5T^1^ | G |  |
|  |  |  |  |  | newbler-isotig06849 | E, G, S |  |
|  |  |  |  |  | Singletons201754 | S |  |
|  |  |  |  |  | Singletons225098^1^ | S |  |
| β-Ketoacyl-ACP synthase I (KASI) | AT5G46290 | 2.3.1.41 | 5 | 5 | 270 | E, G, S | E, G |
|  |  |  |  |  | 4138 | E, G, S | E, G, S* |
|  |  |  |  |  | GZ44Y0R02GEK1V | E |  |
|  |  |  |  |  | newbler-isotig00666 | E, G, S |  |
|  |  |  |  |  | Singletons237695 | E, G, S | E, G, S* |
| β-ketoacyl-ACP synthase II (KASII) | AT1G74960 | 2.3.1.179 | 3 | 2 | 78 | E, G, S |  |
|  |  |  |  |  | newbler-isotig00674 | G |  |
|  |  |  |  |  | Singletons202679 | S |  |
| β-Ketoacyl-ACP synthase III (KASIII) |  | 2.3.1.180 | 2 | 2 | 1809 | E, G, S | G, S* |
|  |  |  |  |  | GZ44Y0R02HCYIS | S |  |
| β-ketoacyl-CoA synthase (KCS) | AT1G04220 | 2.3.1.16 | 25 | 24 | 1638 | E, G, S |  |
|  | AT1G07720 |  |  |  | 3721 | E, G, S | E |
|  | AT1G19440 |  |  |  | 3937 | E, G, S |  |
|  | AT1G25450 |  |  |  | 4332 | E, G, S |  |
|  | AT1G68530 |  |  |  | 4500 | E, G, S | E |
|  | AT2G16280 |  |  |  | 4879 | E, S |  |
|  | AT2G26640 |  |  |  | newbler-isotig01406 | E, G, S |  |
|  | AT2G28630 |  |  |  | newbler-isotig01407 | E, G, S |  |
|  | AT5G43760 |  |  |  | newbler-isotig08080 | G, S |  |
|  |  |  |  |  | Singletons10268 | S |  |
|  |  |  |  |  | Singletons11620 | G, S |  |
|  |  |  |  |  | Singletons11671 | E, S |  |
|  |  |  |  |  | Singletons1231 | G |  |
|  |  |  |  |  | Singletons12560 | E, G |  |
|  |  |  |  |  | Singletons200249 | G |  |
|  |  |  |  |  | Singletons200318 | E |  |
|  |  |  |  |  | Singletons202890 | S |  |
|  |  |  |  |  | Singletons210303 | S |  |
|  |  |  |  |  | Singletons214987 | S |  |
|  |  |  |  |  | Singletons215437 | S |  |
|  |  |  |  |  | Singletons228159 | G, S |  |
|  |  |  |  |  | Singletons230198 | S |  |
|  |  |  |  |  | Singletons232972 | S |  |
|  |  |  |  |  | Singletons233714 | G |  |
|  |  |  |  |  | Singletons5582 | S |  |
| enoyl-CoA reductase (ECR) | AT3G55360 | 1.3.1.8, 1.3.1.38 | 4 | 4 | 3661 | E, G, S |  |
|  |  |  |  |  | newbler-isotig07705 | E, G, S |  |
|  |  |  |  |  | Singletons207910 | E, S |  |
|  |  |  |  |  | Singletons228054 | G, S |  |
| enoyl-ACP reductase (EAR) | AT2G05990 | 1.3.1.9 | 5 | 4 | GZ44Y0R01BJIBO | S |  |
|  |  |  |  |  | newbler-isotig01102 | E, G, S | E, G, S* |
|  |  |  |  |  | newbler-isotig01103 | E, G, S | E, G, S* |
|  |  |  |  |  | Singletons237178 | E, S | E, G, S* |
|  |  |  |  |  | Singletons4323 | S |  |
| fatty acid desaturase (FAD) | AT3G12120 | 1.14.19.4 | 9 | 9 | 2484 | E, G, S |  |
|  | AT4G30950 |  |  |  | 686 | E, G, S |  |
|  | AT5G05580 |  |  |  | 785 | E, G, S |  |
|  |  |  |  |  | newbler-isotig06209 | E, G, S |  |
|  |  |  |  |  | Singletons14713 | G, S |  |
|  |  |  |  |  | Singletons208640 | S |  |
|  |  |  |  |  | Singletons217678 | G |  |
|  |  |  |  |  | Singletons223985 | E, G, S |  |
|  |  |  |  |  | Singletons237933 | S |  |
| fatty acyl-CoA reductase (FAR) | AT3G11980 | 1.2.1.42 | 16 | 7 | 3767 | E, G, S |  |
|  | AT3G56700 |  |  |  | newbler-isotig00011 | E, G, S |  |
|  |  |  |  |  | newbler-isotig00012 | E, G, S |  |
|  |  |  |  |  | newbler-isotig00013 | E, G, S |  |
|  |  |  |  |  | newbler-isotig00014 | E, G, S |  |
|  |  |  |  |  | newbler-isotig00015 | E, G, S |  |
|  |  |  |  |  | newbler-isotig00016 | E, G, S |  |
|  |  |  |  |  | newbler-isotig00017 | E, G, S |  |
|  |  |  |  |  | newbler-isotig00018 | E, G, S |  |
|  |  |  |  |  | newbler-isotig00019 | E, G, S | E, G, S* |
|  |  |  |  |  | newbler-isotig00020 | E, G, S | E, G, S* |
|  |  |  |  |  | newbler-isotig05892 | G, S | E, G* |
|  |  |  |  |  | newbler-isotig07704 | E, G, S |  |
|  |  |  |  |  | newbler-isotig08232 | G, S |  |
|  |  |  |  |  | Singletons12813 | E, G, S |  |
|  |  |  |  |  | Singletons9563 | E, S |  |
| ferredoxin (Fd) | AT1G10960 |  | 6 | 6 | 2186 | E, G, S |  |
|  | AT1G32550 |  |  |  | 1798 | E, G, S |  |
|  | AT1G60950 |  |  |  | 2208 | G, S |  |
|  | AT2G27510 |  |  |  | newbler-isotig04604 | E, G, S |  |
|  | AT4G21090 |  |  |  | newbler-isotig07492 | E, G, S |  |
|  |  |  |  |  | Singletons7228 | S |  |
| long-chain acyl-CoA synthetase (LACS) | AT1G49430 | 6.2.1.3 | 16 | 16 | 764 | E, G, S | S* |
|  | AT1G64400 |  |  |  | 1034 | E, G, S |  |
|  | AT1G77590 |  |  |  | 1145 | E, G, S |  |
|  | AT2G04350 |  |  |  | 1387 | E, G, S | S* |
|  | AT2G47240 |  |  |  | 3124 | E, G, S | E, G* |
|  | AT3G05970 |  |  |  | 3275 | E, G, S |  |
|  | AT4G23850 |  |  |  | Singletons200874 | G |  |
|  | AT5G27600 |  |  |  | Singletons214779 | E, G, S |  |
|  |  |  |  |  | Singletons215457 | S |  |
|  |  |  |  |  | Singletons218538 | G |  |
|  |  |  |  |  | Singletons219463 | E, S |  |
|  |  |  |  |  | Singletons228335 | S |  |
|  |  |  |  |  | Singletons229923 | S |  |
|  |  |  |  |  | Singletons233237 | E, G |  |
|  |  |  |  |  | Singletons236369 | G |  |
|  |  |  |  |  | Singletons236470 | S | E* |
| lysophosphatidic acid acyl transferase (LPAAT) | AT3G18850 | 2.3.1.51 | 4 | 4 | 1662 | E, G, S | E, G, S* |
|  | AT3G57650 |  |  |  | 3268 | E, G |  |
|  | AT4G30580 |  |  |  | Singletons232846 | E, G |  |
|  |  |  |  |  | Singletons236342 | G, S |  |
| stearoyl/acyl-ACP desaturase (SAD) | AT1G43800 | 1.14.19.2 | 13 | 9 | 2020 | E, G, S |  |
|  | AT2G43710 |  |  |  | GZ44Y0R01A6MHH | G | E, G, S* |
|  | AT5G16230 |  |  |  | GZ44Y0R01BMA90 | G | E |
|  |  |  |  |  | newbler-isotig00116 | E, G, S |  |
|  |  |  |  |  | newbler-isotig00117 | E, G, S |  |
|  |  |  |  |  | newbler-isotig00118 | E, G, S |  |
|  |  |  |  |  | newbler-isotig00119 | E, G, S |  |
|  |  |  |  |  | newbler-isotig00120 | E, S |  |
|  |  |  |  |  | newbler-isotig04461 | E, G, S |  |
|  |  |  |  |  | newbler-isotig05612 | E, G, S |  |
|  |  |  |  |  | newbler-isotig05688 | E, G, S |  |
|  |  |  |  |  | newbler-isotig08608 | E, S |  |
|  |  |  |  |  | Singletons10561 | S |  |
| wax ABC transporter (WAT) | AT1G17840 |  | 12 | 12 | newbler-isotig04298 | E, G, S |  |
|  | AT1G51500 |  |  |  | newbler-isotig04359 | E, G, S |  |
|  |  |  |  |  | newbler-isotig05592 | E, G, S |  |
|  |  |  |  |  | newbler-isotig06356 | G, S |  |
|  |  |  |  |  | newbler-isotig06713 | E, G, S |  |
|  |  |  |  |  | Singletons12333 | S |  |
|  |  |  |  |  | Singletons14622 | S |  |
|  |  |  |  |  | Singletons203269 | S |  |
|  |  |  |  |  | Singletons207887 | S |  |
|  |  |  |  |  | Singletons226778 | G |  |
|  |  |  |  |  | Singletons227477 | S | E, S |
|  |  |  |  |  | Singletons233010 | S |  |
| ***Anthocyanin biosynthesis*** | | | | | | | |
| anthocyanidin synthase (ANS) | AT4G22880 | 1.14.11.19 | 10 | 10 | 2465 | E, G, S | E, G, S* |
|  |  |  |  |  | 3231 | E, G |  |
|  |  |  |  |  | GZ44Y0R01APL3N | E |  |
|  |  |  |  |  | GZ44Y0R01B21BU | S |  |
|  |  |  |  |  | GZ44Y0R02HNTZH | S |  |
|  |  |  |  |  | newbler-isotig02973 | E, G, S |  |
|  |  |  |  |  | Singletons215814 | S |  |
|  |  |  |  |  | Singletons216967 | S | E, G |
|  |  |  |  |  | Singletons220670 | S | E, G |
|  |  |  |  |  | Singletons8592 | S | S |
| chalcone isomerase (CHI) |  | 5.5.1.6 | 4 | 4 | 2565 | E, G, S |  |
|  |  |  |  |  | 944 | E, G, S | E, G, S* |
|  |  |  |  |  | 4007 | E, G, S |  |
|  |  |  |  |  | Singletons6677 | S |  |
| chalcone synthase (CHS) | AT5G13930 | 2.3.1.74 | 24 | 24 | 3238 | E, G, S |  |
|  |  |  |  |  | GZ44Y0R01BBKZ1 | E |  |
|  |  |  |  |  | GZ44Y0R01BYV81 | E |  |
|  |  |  |  |  | GZ44Y0R01C5NNR | E |  |
|  |  |  |  |  | GZ44Y0R01CD2L1 | G |  |
|  |  |  |  |  | GZ44Y0R01D7R5W | S |  |
|  |  |  |  |  | GZ44Y0R01DHVW6 | G |  |
|  |  |  |  |  | GZ44Y0R01DJ02S | G |  |
|  |  |  |  |  | GZ44Y0R01DNULR | G |  |
|  |  |  |  |  | GZ44Y0R01EQGKW | G |  |
|  |  |  |  |  | GZ44Y0R01ETOWA | E |  |
|  |  |  |  |  | GZ44Y0R02FPQFA | E |  |
|  |  |  |  |  | GZ44Y0R02I2SA4 | S |  |
|  |  |  |  |  | GZ44Y0R02ILOB0 | S |  |
|  |  |  |  |  | GZ44Y0R02IW86V | E |  |
|  |  |  |  |  | newbler-isotig03069 | E, G, S | S* |
|  |  |  |  |  | newbler-isotig08620 | E, G |  |
|  |  |  |  |  | Singletons10335 | G, S |  |
|  |  |  |  |  | Singletons10448 | S |  |
|  |  |  |  |  | Singletons214170 | S | E, G, S* |
|  |  |  |  |  | Singletons225641 | S |  |
|  |  |  |  |  | Singletons2877 | E, S |  |
|  |  |  |  |  | Singletons404 | S |  |
|  |  |  |  |  | Singletons4532 | G, S |  |
| dihydroflavonol 4-reductase (DFR) | AT4G35420 | 1.1.1.219 | 4 | 3 | 203 | E, G, S |  |
|  | AT5G42800 |  |  |  | 726 | E, G, S | E, G, S* |
|  |  |  |  |  | newbler-isotig01074 | E, G, S | E, G, S* |
|  |  |  |  |  | newbler-isotig08645 | E, G, S |  |
| flavanone 3-hydroxylase (F3H) | AT3G51240 | 1.14.11.9 | 11 | 10 | 324 | E, G, S | E, G, S* |
|  |  |  |  |  | GZ44Y0R02GMWOG | S |  |
|  |  |  |  |  | GZ44Y0R02JWMNU | S |  |
|  |  |  |  |  | newbler-isotig01419 | E, G, S |  |
|  |  |  |  |  | Singletons12194 | E, G, S |  |
|  |  |  |  |  | Singletons14657 | S |  |
|  |  |  |  |  | Singletons14720 | S | G* |
|  |  |  |  |  | Singletons207688 | S |  |
|  |  |  |  |  | Singletons218872 | S | G, S* |
|  |  |  |  |  | Singletons221365 | S |  |
|  |  |  |  |  | Singletons233199 | G, S |  |
| flavonoid 3'-hydroxylase (F3'H) | AT5G07990 | 1.14.13.21 | 7 | 7 | GZ44Y0R01A9KBI | G |  |
|  |  |  |  |  | GZ44Y0R01BQ6J0 | S |  |
|  |  |  |  |  | GZ44Y0R01C94PD | S |  |
|  |  |  |  |  | Singletons202051 | S |  |
|  |  |  |  |  | Singletons209684 | G, S |  |
|  |  |  |  |  | Singletons214394 | S |  |
|  |  |  |  |  | Singletons218097 | S |  |
| flavonoid 3',5'-hydroxylase (F3'5'H) |  | 1.14.13.88 | 0^2^ | 0 |  |  |  |
| UDP-glucose flavonol 3-O-glucosyltransferase (UF3GT) |  | 2.4.1.91 | 1 | 1 | 3671 | E, G, S |  |

^1^The assignment of these four transcripts is ambiguous, since they matched both EC 1.1.1.35 and EC 4.2.1.17 in EC annotation.

^2^No transcripts were found for F3’5’H using our criteria for candidate transcript identification. When using BLASTN searches of known *F3’5’H* sequences (GenBank accessions ACC59773.2, AEB96145.1, DQ148458.1, HQ412560, and XM_003627258.1) as queries, four orchid transcripts (23, Singletons6900, newbler-isotig00307, newbler-isotig00308) are identified as homologous at an e-value < 1E-10. However, all of these transcripts have more significant BLASTN and BLASTX hits to other sequences in the NCBI nr database.
